# Cross‐sectional trends in HIV prevalence among pregnant women in Botswana: an opportunity for PrEP?

**DOI:** 10.1002/jia2.25892

**Published:** 2022-03-24

**Authors:** Andrew Kapoor, Aamirah Mussa, Modiegi Diseko, Gloria Mayondi, Judith Mabuta, Mompati Mmalane, Joseph Makhema, Chelsea Morroni, Shahin Lockman, Rebecca Zash, Roger Shapiro

**Affiliations:** ^1^ Division of Infectious Diseases Beth Israel Deaconess Medical Center Boston Massachusetts USA; ^2^ Harvard Medical School Boston Massachusetts USA; ^3^ Botswana Harvard AIDS Institute Partnership Gaborone Botswana; ^4^ Centre for Reproductive Health University of Edinburgh Edinburgh UK; ^5^ Department of Immunology and Infectious Diseases Harvard T. H. Chan School of Public Health Boston Massachusetts USA; ^6^ Division of Infectious Diseases Brigham and Women's Hospital Boston Massachusetts USA

**Keywords:** HIV surveillance, pregnancy, HIV pre‐exposure prophylaxis, Botswana, HIV epidemiology, HIV prevention

## Abstract

**Introduction:**

Young women in sub‐Saharan Africa are at particularly high risk of HIV acquisition. Recent shifts towards “test and treat” strategies have potential to reduce transmission in this age group but have not been widely studied outside of clinical trials. Using data from nationwide surveillance among pregnant women in Botswana, where a “test and treat” program was implemented in 2016, we describe trends in HIV prevalence over time and highlight opportunities for targeted prevention.

**Methods:**

The Tsepamo study abstracted data from obstetric records of all women delivering at eight government hospitals in Botswana between 2015 and 2019, accounting for 45% of all births in the country (*n* = 120,755). We used a stratified analysis to identify prevalence trends and evaluated decreases in HIV prevalence over time using the Cochrane–Armitage test for linear trend. A multivariable logistic regression analysis was also performed to identify factors associated with declines in HIV prevalence.

**Results:**

Overall HIV prevalence was 24.1% among 120,755 women who delivered during the study period. Prevalence differed by site of delivery, ranging from 16.1% to 28.2%, and increased markedly with age. Lower educational attainment (adjusted odds ratio [aOR] = 3.28; 95% confidence interval [CI] 3.07–3.50) and being unmarried (aOR = 1.98; 95% CI 1.88–2.08) were associated with HIV infection. HIV prevalence was 10.0% with a first pregnancy, 21.0% with a second and 39.2% with a third or greater (aOR = 2.20; for any prior pregnancy; 95% CI 2.10–2.29). The same age‐adjusted trends were seen when data were limited to women aged 15–24, with a two‐ to three‐fold increase in HIV prevalence between a first and third pregnancy. Prevalence decreased linearly during the 5‐year study period from 25.8% to 22.7% (*p* <0.001). Among age‐specific strata, the greatest absolute decline occurred in those aged 35–39, with an 8.7% absolute decrease in HIV prevalence from 2015 to 2019. Minimal declines were seen in those 15–24, with a decrease of only 1.5% over the same period.

**Conclusions:**

While overall trends in Botswana show HIV prevalence declining among pregnant women, prevalence among the youngest age group has remained stagnant. Preventative interventions utilizing pre‐exposure prophylaxis should be prioritized during the high‐risk period surrounding a woman's first pregnancy.

## 1 INTRODUCTION

Globally, the incidence of HIV has been declining due to improvements in the access and affordability of antiretroviral therapy (ART). Though this trend is encouraging, sub‐Saharan Africa still accounts for 70% of all new HIV infections despite representing only 10% of the global population [1, 2]. In recent years, it has become clear that young women bear the burden of this risk in sub‐Saharan Africa particularly those aged 15–24 [2, 3]. To address this burden, many high prevalence countries have adopted universal test and treat (UTT) strategies aimed at improving ART coverage and community‐level viral suppression [4, 5, 6, 7]. Several large population‐based trials have shown UTT to be successful in reducing HIV incidence but the population‐level impacts of these programs have not been well studied outside of clinical trials [4].

Given the epidemiological importance of adolescents and young women, it is critical to have granular data looking at HIV trends within this sub‐group [8]. Collecting such data has been challenging in low‐and middle‐income countries (LMICs) as prospective longitudinal cohorts are costly, time‐consuming and require significant human capital [5]. However, LMICs already have established networks of antenatal clinics and birth outcome databases that collect data on pregnant women [9, 10]. This pre‐existing infrastructure offers a cost‐effective way to capture and study HIV trends and already forms the basis for global estimates [11]. Botswana in particular has emerged as a unique place to study HIV in pregnancy given the availability of free antenatal care, free HIV testing and widespread uptake of ART (including UTT since May 2016) [12]. In addition, it has a stable total fertility rate of 2.8, 95% of women deliver in hospital and the uptake of HIV testing during pregnancy has been consistently >95% over the past decade [13, 14]. Collectively, these factors have mitigated major barriers to providing high‐quality HIV care to pregnant women, but it is not yet known if these strategies or the implementation of UTT has translated into decreasing HIV prevalence.

Using data from nationwide surveillance among pregnant women in Botswana (the Tsepamo Study), we set out to describe trends in HIV prevalence over time among pregnant women between 2015 and 2019 at eight hospital sites. We then compared these results across time, age groups and regions to better understand the trends and associated factors shaping the dynamics of the HIV epidemic in Botswana. Our results provide epidemiologic insight into how country‐level interventions, including UTT, have impacted HIV prevalence among pregnant women, and highlight the optimal window for targeting pre‐exposure prophylaxis (PrEP) interventions in Botswana.

## METHODS

We conducted a retrospective serial cross‐sectional study using data from the Tsepamo Study [15], which performs birth outcomes surveillance at 18 government hospitals in Botswana. The Tsepamo database is a nationally representative dataset that captures data from the obstetrical records of women in Botswana who deliver live or stillborn infants in participating maternity wards. For this analysis, we utilized data from the eight original Tsepamo sites, which had complete data from January 2015 to December 2019, and accounted for approximately 45% of all births in the country during this period. Ten sites were excluded given that they were added between 2018 and 2019, and did not have complete data covering the study period of interest. The study sites included were comprised of two tertiary referral hospitals, five district hospitals and one primary‐level hospital. Analyses were performed using SAS 9.4 University Edition and Stata (Version 16, StataCorp, College Station, TX). Ethics approval for this study was granted by the Botswana Human Research and Development Division and by the institutional review board of Harvard T. H. Chan School of Public Health. Informed consent was not required because records were deidentified and the study was observational.

Our main exposures of interest were maternal age, calendar time and hospital location. Maternal age was documented at delivery and categorized into four strata (<15, 15–24, 25–35 and >35). However, narrower age strata using increments of 5 years were used in the descriptive analysis to allow for more granular trends to be characterized. Calendar time was analysed categorically and stratified by year to allow for the assessment of annual trends. Hospital location served as a proxy for maternal home district and covered both urban and rural areas of Botswana. Additional demographic data, including marital status, education, gravida and occupation, were used as measures of socio‐economic status and included in the regression model as categorical variables to identify factors associated with HIV infection. The primary outcome was HIV status at delivery, which was used to calculate prevalence. Maternal HIV status was obtained directly from the obstetric card and in most cases, was confirmed by additional HIV treatment records and maternal confirmation at the maternity ward. A subset of diagnoses were also verified through direct access to Botswana's national HIV laboratory system if there were discrepancies. HIV diagnosis in Botswana is generally made using dual enzyme immunoassay testing, and HIV RNA testing by PCR is also performed in the context of treatment. At each site, research assistants abstracted de‐identified data, including the primary outcome and covariates, from maternal obstetrical cards at the time of discharge from the postnatal ward and entered into the Tsepamo database. Maternal deaths during delivery were not included in the dataset.

We used descriptive stratified analyses to identify prevalence trends by age, calendar year, gravida and location. Our unadjusted stratified analysis included a quantitative assessment of trends using Cochran–Armitage trend testing to help identify significant decreases in HIV prevalence over time. Subsequently, we used a multivariable logistic regression model, where HIV status at delivery was used as the outcome variable, to estimate the adjusted odds ratios of being HIV positive while controlling for age, calendar year, delivery site, marital status, education, gravida and employment. The model was also used to verify significant trends in HIV prevalence by age, calendar year and location in the descriptive analysis. A complete case analysis was performed and collinearity testing between variables was also conducted to ensure that variables with a variance inflation factor (VIF) greater than five were not included in the regression model. Sensitivity analyses were also performed to determine whether women with a missing or undisclosed HIV serostatus impacted the direction of associations by assuming that women with missing HIV serostatus were either all HIV positive or all HIV negative.

## RESULTS

Over the study period between 1 January 2015 and 31 December 2019, all maternal/birth records available were abstracted and a total of 121,710 records from across the eight hospital sites were reviewed. We excluded 955 (0.7%) due to non‐disclosure of HIV status and thus, a total of 120,755 delivering women were included in the analysis; 29,115 (24%) were HIV positive and 91,640 (76%) were HIV negative. Maternal HIV status was documented in >99% of records along with their demographic data. The distribution of deliveries by age, calendar year, occupational status, education and delivery site is shown in Table 1, stratified by HIV serostatus. The study sample captured a significant proportion of adolescents and young women between the ages of 15 and 24, who comprised 41% of the overall cohort. The majority of women presenting for delivery reported being unmarried (89%), having a secondary‐level education or lower (77.2%) and without a regular source of personal income (student, unemployed or working at home) (65%). Concerning gravida, 36% of women captured were presenting with a first pregnancy and 64% with a second or greater. The number of deliveries by site varied but was proportional to hospital size with the two tertiary hospitals (Princess Marina and Nyangabgwe) contributing 48% of the total number of deliveries. The absolute number of deliveries per calendar year was consistent across the 5‐year study period with each year accounting for approximately 20% of the total sample (Table 1).

**Table 1:** Baseline demographic characteristics of study participants at delivery stratified by HIV serostatus

	Total *N* = 120,755	HIV negative *N* = 91,640	HIV positive *N* = 29,115
Age	*n* (%)	*n* (%)	*n* (%)
Under 15	157 (0.13)	153 (97.5)	4 (2.6)
15–24	49,536 (41.0)	43,721 (88.3)	5815 (11.7)
25–35	55,789 (46.2)	39,829 (71.4)	15,960 (28.6)
35 and older	15,218 (12.6)	7891 (51.9)	7327 (48.2)
Unknown	55 (0.1)	46 (83.6)	9 (16.4)
Calendar time			
2015	23,405 (19.4)	17,364 (74.2)	6041 (25.8)
2016	24,280 (20.1)	18,357 (75.6)	5923 (24.4)
2017	24,517 (20.3)	18,536 (75.6)	5981 (24.4)
2018	24,298 (20.1)	18,635 (76.7)	5663 (23.3)
2019	24,255 (20.1)	18,748 (77.3)	5507 (22.7)
Marital status			
Single	104,094 (89.0)	78,735 (75.6)	25,359 (24.4)
Married	12,432 (10.6)	9814 (78.9)	2618 (21.1)
Widowed/divorced	383 (0.3)	216 (56.4)	167 (43.6)
Unknown	3846 (3.2)	2875 (74.8)	971 (25.3)
Occupation			
Student	7874 (6.8)	7180 (91.2)	694 (8.8)
Unemployed/housewife	67,108 (58.0)	50,522 (75.3)	16,586 (24.7)
Salaried/paid employment	40,743 (35.2)	30,196 (74.1)	10,547 (25.9)
Unknown	5030 (4.2)	3742 (74.4)	1288 (25.6)
Education			
Primary or below	8788 (7.5)	5318 (60.5)	3470 (39.5)
Secondary or equivalent	82,197 (69.7)	60,824 (74.0)	21,373 (26.0)
Tertiary or equivalent	26,906 (22.8)	23,425 (87.1)	3481 (12.9)
Unknown	2864 (2.4)	2073 (72.4)	791 (27.6)
Gravida			
Primigravida	42,949 (35.6)	38,654 (90.0)	4295 (10.0)
Multigravida	77,806 (64.4)	52,986 (68.1)	24,820 (31.9)
Delivery site			
Princess Marina Hospital (tertiary, south)	32,187 (26.7)	25,094 (78.0)	7093 (22.0)
Nyangabgwe (tertiary, east)	25,828 (21.4)	18,548 (71.8)	7280 (28.2)
Molepolole (district, south)	15,587 (12.9)	12,125 (77.8)	3462 (22.2)
Ghanzi (primary, west)	4339 (3.6)	3639 (83.9)	700 (16.1)
Maun (district, north)	14,669 (12.2)	11,141 (76.0)	3528 (24.1)
Serowe (district, central)	12,247 (10.1)	9167 (74.9)	3080 (25.2)
Mahalapye (district, central)	8857 (7.3)	6772 (76.5)	2085 (23.5)
Selebi‐Phikwe (district, east)	7041 (5.8)	5154 (73.2)	1887 (26.8)

Abbreviation: *N*, number of women.

### Age and time‐related trends

**Figure 1:**
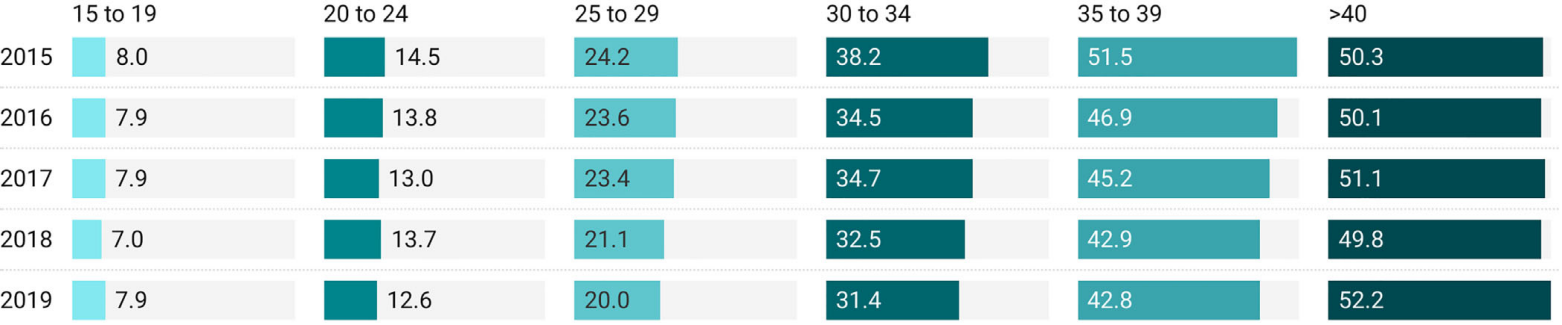
HIV prevalence by age category among women in the Tsepamo study.

**Figure 2:**
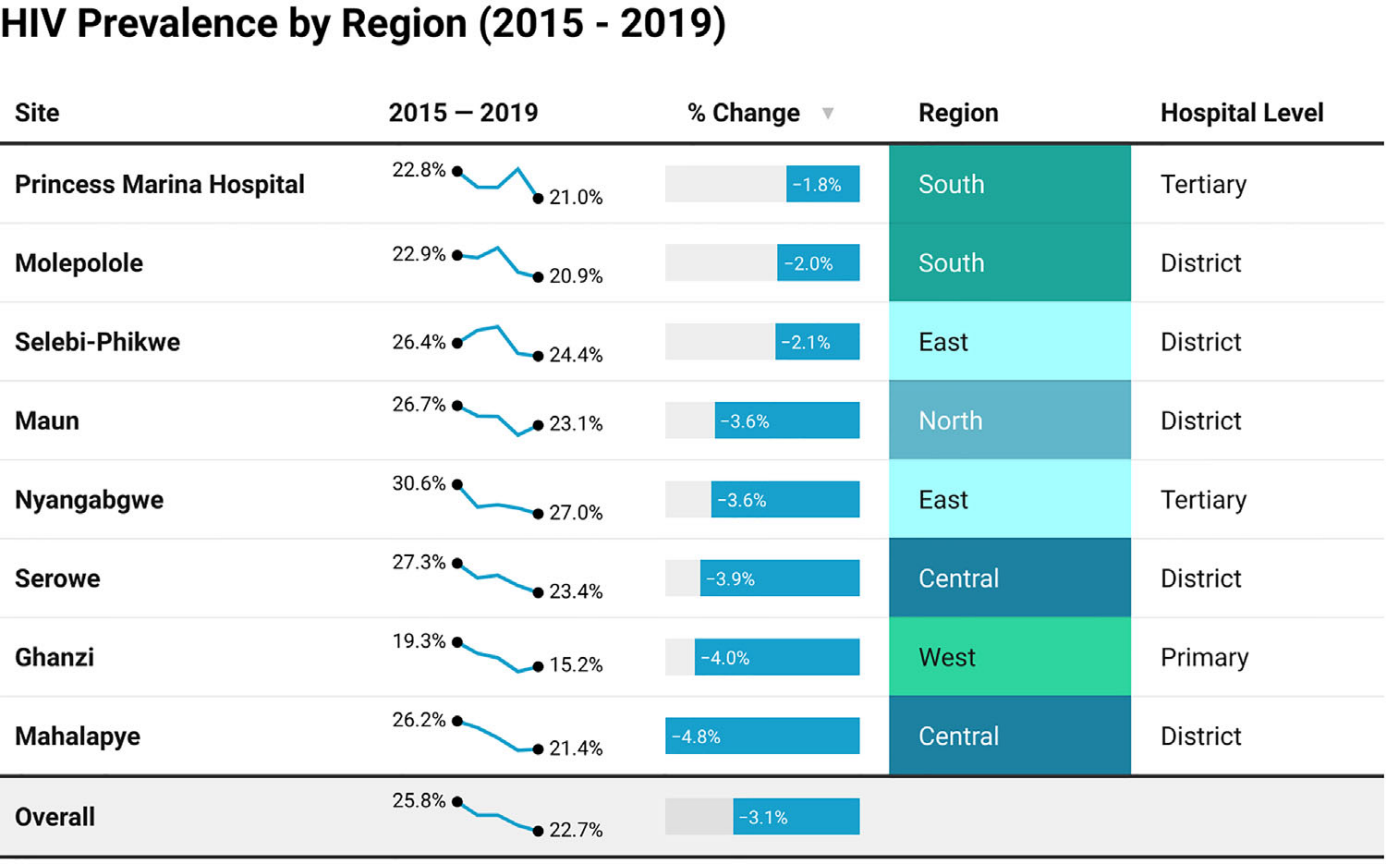
Overall and regional trends in HIV prevalence among women in the Tsepamo study.

When examining the overall prevalence prior to the initiation of UTT by age group, women 15–24 (*n* = 49,536) had a high overall HIV prevalence of 11.7%, which increased markedly with age. Those who presented over the age of 40 (*n* = 4485) had extremely high HIV prevalence approximating 50% (Figure 1). The overall prevalence of HIV decreased linearly during the 5‐year study period, during which time UTT was introduced and scaled up, from 25.8% in 2015 to 22.7% in 2019 (*p* <0.001) demonstrating a statistically significant decline of 3.1% (Figure 2) using Cochrane–Armitage trend testing. When looking at prevalence post UTT among age‐specific strata, declines over time were seen in all age groups except those greater than 40 years old, where there was a slight increase of 1.9% (*n* = 4485) over the 5‐year study period. When age‐related declines were looked at granularly in 5‐year increments, absolute declines ranged from 0.1% among those 15–19 to 8.7% in those aged 35–39 between 2015 and 2019 (Figure 1). After the implementation of UTT, young women and adolescents aged 15–24 saw lower declines at 1.5% compared with those 25 years and older, where declines were 4.9%. This reflects a three‐fold lower relative decline in HIV prevalence among young women and adolescents compared with women 25 and older. The most minimal declines post UTT of 0.1% were seen among the very youngest group of women aged 15–19.

### Site‐related trends

The eight study sites represented two tertiary hospitals, five district hospitals and one primary‐level hospital setting. The prevalence of HIV varied significantly by the site of delivery ranging from 19.3% to 30.6% at the start of the study period, representing a difference of up to 11.3%. At study completion, prevalence declined overall and ranged between 16.1% and 28.2% with a similar spread of 12.1%. All sites saw statistically significant net declines in prevalence, which again varied by site, ranging from 1.8% to almost 5%. The two most southern sites, Princess Marina Hospital and Molepolole, seemed to have the lowest net decreases in prevalence at approximately 2%. Whereas the central and western sites, including Serowe, Ghanzi and Mahalapye, appeared to have the more robust declines in the range of 4–5%. There appeared to be no clear association between the service level of each hospital and the overall net declines seen in prevalence (Figure 2).

### Pregnancy‐related trends

**Figure 3:**
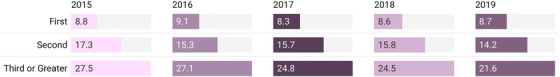
HIV prevalence by number of prior pregnancies among women aged 15–24 within the Tsepamo study.

Overall HIV prevalence by gravida showed significant increases with subsequent pregnancy from 10.0% with a first pregnancy, to 21.0% with a second pregnancy, and 39.2% with a third or greater pregnancy. These increases in prevalence represent a two‐fold difference between a first and second pregnancy and a four‐fold increase between a first and third pregnancy. When looking specifically at women aged 15–24 (*n* = 5395), the same age‐adjusted trends in HIV prevalence were seen, with 8.7% among first pregnancies, 15.7% with a second and 25.1% with a third or greater pregnancy, thus, demonstrating a persistent two‐ to three‐fold increase between pregnancies, even among younger women (Figure 3).

### Regression analysis

The unadjusted analysis showed that educational attainment, gravida and marital status had the strongest associations with HIV infection (Table 2). Those with primary education or below had higher odds of being HIV positive (unadjusted odds ratio [uOR] = 4.39; 95% confidence interval [CI] 4.15–4.64), as well as those who were unmarried (uOR = 1.20; 95% CI 1.14–1.25) and those with multiple pregnancies (uOR = 4.21; 95% CI 4.06–4.36). These findings were preserved in the adjusted analysis but associations were slightly attenuated when controlling for demographic factors (Table 2). In these adjusted models, lower educational attainment (adjusted odds ratio [aOR] = 3.28; 95% CI 3.07–3.50) and being unmarried (aOR = 1.98; 95% CI 1.88–2.08) remained associated with HIV infection, as did being multigravida (OR = 2.20; 95% CI 2.10–2.29).

**Table 2:** Regression analysis of factors associated with HIV infection among women in the Tsepamo study

	Total *N* = 112,332	HIV negative *N* = 85,329	HIV positive *N* = 27,003	uOR (95% CI)	*p* Value	aOR (95% CI)	*p* Value
Age	*n* (%)	*n* (%)	*n* (%)				
Under 15	139 (0.1)	135 (97.1)	4 (2.9)	0.03 (0.01–0.09)	<0.001	0.04 (0.02–0.12)	<0.001
15–24	46,016 (41.0)	40,621 (88.3)	5395 (11.7)	0.14 (0.14–0.15)	<0.001	0.19 (0.18–0.20)	<0.001
25–35	52,050 (46.3)	37,243 (71.6)	14,807 (28.5)	0.43 (0.41–0.45)	<0.001	0.46 (0.44–0.48)	<0.001
35 and older	14,127 (12.6)	7330 (51.9)	6797 (48.1)	Ref	Ref	Ref	Ref
Calendar time							
2015	21,608 (19.2)	16,061 (74.3)	5547 (25.7)	Ref	Ref	Ref	Ref
2016	22,658 (20.2)	17,119 (75.6)	5539 (24.5)	0.94 (0.90–0.98)	0.003	0.95 (0.91–1.00)	0.033
2017	22,935 (20.4)	17,353 (75.7)	5582 (24.3)	0.93 (0.89–0.97)	0.001	0.92 (0.88–0.97)	0.001
2018	22,574 (20.1)	17,342 (76.8)	5232 (23.2)	0.87 (0.84–0.91)	<0.001	0.85 (0.81–0.89)	<0.001
2019	22,557 (20.1)	17,454 (77.4)	5103 (22.6)	0.85 (0.81–0.88)	<0.001	0.82 (0.78–0.86)	<0.001
Marital status							
Single	100,039 (89.1)	75,723 (75.7)	24,316 (24.3)	1.20 (1.14–1.25)	<0.001	1.98 (1.88–2.08)	<0.001
Married	11,930 (10.6)	9404 (78.8)	2526 (21.2)	Ref	Ref	Ref	Ref
Widowed/divorced	363 (0.3)	202 (55.7)	161 (44.4)	2.97 (2.40–3.67)	<0.001	2.60 (2.08–3.26)	<0.001
Occupation							
Student	7607 (6.8)	6939 (91.2)	668 (8.8)	0.29 (0.27–0.32)	<0.001	0.91 (0.83–0.99)	0.029
Unemployed/housewife	65,083 (57.9)	48,990 (75.3)	16,093 (24.7)	Ref	Ref	Ref	Ref
Salaried/paid employment	39,642 (35.3)	29,400 (74.2)	10,242 (25.8)	1.06 (1.03–1.09)	<0.001	0.95 (0.92–0.98)	0.002
Education							
Primary or below	8051 (7.2)	4805 (59.7)	3246 (40.3)	4.54 (4.29–4.81)	<0.001	3.28 (3.07–3.50)	<0.001
Secondary or equivalent	78,342 (69.7)	57,942 (74.0)	20,400 (26.0)	2.37 (2.28–2.46)	<0.001	2.36 (2.26–2.47)	<0.001
Tertiary or equivalent	25,939 (23.1)	22,582 (87.1)	3357 (12.9)	Ref	Ref	Ref	Ref
Gravida							
Primigravida	40,156 (35.8)	36,141 (90.0)	4015 (10.0)	Ref	Ref	Ref	Ref
Multigravida	72,176 (64.3)	49,188 (68.2)	22,988 (31.9)	4.21 (4.06–4.36)	<0.001	2.20 (2.10–2.29)	<0.001
Delivery site							
Princess Marina (tertiary, south)	29,571 (26.3)	23,105 (78.1)	6466 (21.9)	Ref	Ref	Ref	Ref
Nyangabgwe (tertiary, north)	24,219 (21.6)	17,436 (72.0)	6783 (28.0)	1.39 (1.34–1.45)	<0.001	1.46 (1.40–1.52)	<0.001
Molepolole (district, south)	14,497 (12.9)	11,297 (77.9)	3200 (22.1)	1.01 (0.96–1.06)	0.621	0.94 (0.89–0.99)	0.019
Ghanzi (primary, west)	3620 (3.2)	3021 (83.5)	599 (16.6)	0.71 (0.65–0.78)	<0.001	0.61 (0.55–0.67)	<0.001
Maun (district, north)	13,514 (12.0)	10,297 (76.2)	3217 (23.8)	1.12 (1.06–1.17)	<0.001	1.01 (0.97–1.07)	0.640
Serowe (district, mid)	11,538 (10.3)	8633 (74.8)	2905 (25.2)	1.20 (1.14–1.26)	<0.001	1.16 (1.10–1.22)	<0.001
Mahalapye (district, mid)	8578 (7.6)	6557 (76.4)	2021 (23.6)	1.10 (1.04–1.17)	0.001	1.04 (0.97–1.10)	0.255
Selebi‐Phikwe (district, north)	6795 (6.1)	4983 (73.3)	1812 (26.7)	1.30 (1.22–1.38)	<0.001	1.37 (1.29–1.47)	<0.001

Abbreviations: aOR, adjusted odds ratio; Ref, referent category used for comparisons; uOR, unadjusted odds ratio.

Collinearity testing identified no evidence of high collinearity between age and gravida (VIF = 1.4). Therefore, both variables were retained in the regression model. In sensitivity analyses, no differences in associations were noted when assuming women with unknown HIV serostatus (0.7%) were either all HIV‐positive or all HIV‐negative women.

## DISCUSSION

Using data from nationwide surveillance among pregnant women in Botswana delivering between 2015 and 2019, we demonstrated a 3.1% decline in total HIV prevalence, with minimal decline among those 15–24. Our data also highlight high prevalence at the time of a second pregnancy, even when adjusting for age, suggesting the time after a first pregnancy may be an ideal window for targeting PrEP interventions among young women in Botswana.

The overall decline in prevalence coincided with the period following UTT introduction in 2016 and aligns with other population‐based studies looking at HIV incidence and prevalence in Botswana [5, 7, 16]. The Botswana Combination Prevention Project (BCPP) was a 30‐community randomized trial that occurred from 2013 to 2018 throughout Botswana and reported a 30% overall decline in incidence in an enhanced UTT setting. Interestingly, the findings from BCPP are consistent with prevalence changes observed in our study during a similar time period [17, 18, 19].

Although the aggregate prevalence trend seen among all pregnant women in the study is encouraging, the static prevalence among the 15–24 age group is concerning but also consistent with data from other studies. HIV prevalence among this youngest group is most likely to mirror incidence, and several recent studies – including BCPP – have highlighted the disproportionally high incidence rates among young women in sub‐Saharan Africa [2, 3, 5, 8]. In BCPP, 16‐ to 24‐year‐old women had the highest reported incidence, mirroring the concerns raised by our dataset [5]. Our results are also consistent with studies conducted among non‐pregnant youth in sub‐Saharan Africa and underscore the ongoing challenge of reducing infection rates among this group [4].

More encouraging trends were seen among women aged 25–39, where consistent declines in HIV prevalence were seen ranging from 5% to almost 9% across this demographic. The cross‐sectional nature of our study limits our ability to infer causes, but the ramp‐up of HIV testing, linkages to care and prevention efforts, which occurred in Botswana in the early 2010s, followed by the adoption of UTT in 2016, are likely to be major contributors. As expected, HIV prevalence increased by age cohort, and among women over 40 prevalence reached 52%. This is likely explained by the effects of ART and the resultant survivor effect, allowing women to live longer and remain fertile despite chronic infection, but also points to ongoing risk for HIV acquisition throughout adulthood. While improvements in survival with ART are undoubtedly positive, the staggering prevalence among older pregnant women highlights how much can be gained from early targeted prevention measures.

The significant rise in HIV prevalence observed with an increasing number of pregnancies underscores the need for new preventative strategies. The high incidence of HIV among women 16–24 years old also highlights the added concern that acute infection may occur during pregnancy, greatly increasing the potential risk of vertical transmission [20, 21]. Our findings show the number of pregnancies to be strongly associated with HIV infection. Even in the adjusted analysis, gravida conferred a two‐fold increase in the odds of having HIV. Data from other cohorts in sub‐Saharan Africa have also found similar trends between pregnancy and HIV risk, and several hypotheses have been proposed [22]. Both behavioural and biological factors have been postulated though it remains unclear if there are also additional risk factors applicable to non‐pregnant women [23]. Previous studies have shown that lower condom use during pregnancy, intergenerational relationships and a rise in sexual partners outside of relationships during pregnancy may play a role [22]. Additionally, hormonal changes affecting the genital tract mucosa or immune response during pregnancy may result in women being more susceptible to HIV infection [24]. Collectively, these data highlight that there are many opportunities for prevention. Although deploying preventative measures prior to high‐risk behaviours is ideal, engaging women during a first pregnancy and in the immediate postpartum period may also be effective, and represents another critical opportunity to intervene.

Advances in PrEP delivery may offer new avenues for prevention among this group. The efficacy of PrEP in men who have sex with men is well documented but studies among adolescents and resource‐limited settings have been less favourable [25, 26, 27, 28]. Adherence and persistence have been identified as barriers among young women in sub‐Saharan Africa and both have significantly limited the potential impact of oral PrEP in this context [29]. However, second‐generation formulations of PrEP that are longer acting have demonstrated the ability to overcome these challenges [30]. A recent trial of long‐acting cabotegravir among cisgender African women found it reduced HIV infection by 89% compared with oral PrEP [31, 32]. Such favourable results, in combination with the marked increase in risk between pregnancies, present an opportunity for impactful intervention. Emerging data have demonstrated that long‐acting PrEP is safe in both pregnant and breastfeeding women, though longer‐term surveillance is urgently needed [33, 34].

A pipeline of newer agents, including lenacapavir, islatrivir and multi‐affinity antibodies, also hold promise for prevention among this group of women. The success of recent trials examining the efficacy of long‐acting PrEP among women in sub‐Saharan Africa underscores its potential as an impactful intervention, and our data suggest that during and immediately after a first pregnancy may be an opportune time to offer PrEP services. To illustrate the potential impact of such a strategy, we estimated that if universal uptake was achieved, the prevalence of HIV among pregnant women could be reduced from 23% to 10% assuming all infections could be prevented between a first and second pregnancy. Though high levels of uptake would be challenging, if even half of women connected with antenatal services initiated long‐acting PrEP, a reduction of 5–6% would be achievable. Linking antenatal services and PrEP delivery may be a practical approach to decreasing HIV prevalence with potential to double the impact demonstrated by our study, which accounted for reductions attributable to all population‐level interventions over a 5‐year period.

The main strength of this analysis is that it leverages the large Tsepamo birth outcomes database with coverage of over 45% of births in Botswana. It draws upon routinely collected data resulting in a largescale, complete and unbiased database of pregnant women. The large sample in this database allows for more nuanced assessments of age‐related, geographic and demographic trends. It further enables prevalence estimates even among small subgroups to be made with precision, helping uncover both macro‐ and micro‐level changes in Botswana's HIV epidemic. Using a birth outcomes database also allowed this study to be low‐cost and feasible. This is an important advantage particularly in LMICs, where the alternative is to conduct large cohort studies, which are resource‐intensive and time‐consuming. Complete antenatal databases with minimal missing data provide an opportunity to inform both pregnancy‐related and national‐level trends in HIV.

Despite these strengths, the cross‐sectional design of this study limits our ability to determine which specific interventions or policies contributed directly to our observed trends. Although the major policy focus in Botswana during the 5‐year study period was the scale‐up of UTT, it is possible that other local, regional or national programs also contributed to declines in HIV prevalence. Interventions, such as voluntary circumcision and population‐based HIV testing, have been stable in Botswana, but the rollout of dolutegravir during this timeframe may have had some potential impact. Additionally, to maintain anonymity for women, individual women are not identifiable discreetly within the database. Thus, it is possible that women could have appeared more than once in the dataset. Our very large sample sizes help to significantly mitigate this impacting the overall prevalence estimates and focusing on prevalence by year prevents women from appearing more than once in comparative estimates. Though this is a noteworthy limitation, our study focus was on the estimation of prevalence rather than incidence, where this issue would have been more limiting.

In addition, given that our study was retrospective, we were not able to add variables and were limited by any missing or incomplete data. Although age, occupation, educational attainment and marital status are well‐established predictors for HIV acquisition, an expanded number of variables may have been informative. The study sites were also pre‐determined and while they covered almost 50% of births in the country, they did not include a large number of small primary centres or the very small number of home births in Botswana. Finally, although the database is large, it does not include all births and specifically does not include those who seek care in the private sector. This is a very small subset of young women but their HIV risk may differ from our cohort. Despite these limitations, we believe the data remain very useful in identifying important national‐level trends.

## CONCLUSIONS

While overall trends in Botswana show HIV prevalence is declining among pregnant women, prevalence among the youngest age groups has remained stagnant. This group of adolescents and young women bear the highest risk of incident HIV infection, highlighting the need for new preventative strategies to address their risk. Our study demonstrated that HIV prevalence doubles between pregnancies and, therefore, the period between pregnancies represents an opportune time to intervene with PrEP. Recent therapeutic advances in long‐acting PrEP have demonstrated extraordinary efficacy among African women in preventing HIV infection. This positions long‐acting PrEP agents as a priority prevention tool that can be leveraged during or after a first pregnancy to prevent the maximal number of HIV infections in adolescents and young women.

## COMPETING INTERESTS

The authors declare no competing interests.

## AUTHORS’ CONTRIBUTIONS

AK, AM, RS and RZ conceptualized this analysis. AK and AM analysed the data and prepared the manuscript, which was critically reviewed by all the listed authors. All authors have read and approved the final manuscript.

## FUNDING

The Tsepamo study was funded by the U.S. National Institutes of Health Funding (NIH/NICHD R01HD080471, R01HD095766 and K23 HD088230). The funding source had no role in the design and conduct of the study; data collection, analysis, reporting; and decision to submit the manuscript for publication.

## Data Availability

The data that support the findings of this study are available on request from the corresponding author, AK. The data are not publicly available given the research database contains data that could compromise the privacy of research participants.
